# Supplementary material for: A protocol of a pilot randomised trial (Action-RESPOND) to support rural and regional communities with implementing community-based systems thinking obesity prevention initiatives
Source: PLoS One. 2024 May 2;19(5):e0302047. doi: 10.1371/journal.pone.0302047 (PMC11065269; doi:10.1371/journal.pone.0302047)
Supplement: S1 File — (DOCX) [file pone.0302047.s002.docx]

| **DEAKIN UNIVERSITY HUMAN RESEARCH ETHICS COMMITTEE**  **PROJECT DESCRIPTION/PROTOCOL** |  |
| --- | --- |

**Instructions for preparing the project description/protocol**

1. The purpose of the Project Description is to provide the scientific and academic background and context of a research project.
2. A Project Description is a **mandatory** component of a submission using the Human Research Ethics Application (HREA).
3. The section headings in this Project Description template represent a structure for presentation of information about a research project that meets the needs of an ethics review body.
4. Not all headings or sub-headings in this template are relevant for each research project. Where a question is not relevant please enter NA into the response box. Please do not delete the question.
5. Researchers may use visual aids embedded in the project description/protocol to assist in describing their project where appropriate (e.g. images, videos etc.).
6. Submissions of clinical trial proposals may use alternative protocol templates, such as the [SPIRIT statement](http://www.spirit-statement.org/).
7. Researchers may choose to submit an existing document (such as a protocol or project description that has already been developed) instead of developing a new document.
8. If researchers choose to submit an existing document instead of using one of the templates provided, they may need to provide indications to the ethics review body of where in the submitted document the content corresponding to the relevant fields in the template are located.
9. There is no need to duplicate information in the HREA into the Project Description or vice versa.
10. Language that is understandable to non-technical reviewers should be used.

**COVID-19**

All research must comply with current COVID-19 restrictions, as well as with Deakin’s [COVIDSafe Management Plan](https://deakin365.sharepoint.com/sites/CampusReactivation/SitePages/Being-COVIDSafe.aspx). Any activities considered as having high COVID-19 risk (e.g. requiring safety measures over and above the COVIDSafe Management Plan and risks covered by the general requirements of entry to campus) must have an approved [COVIDSafe Activity Plan](https://deakin365.sharepoint.com/sites/CampusReactivation/SitePages/Being-COVIDSafe.aspx) in place. This includes any on-campus research involving a face-to-face element, as well as off-site research (e.g., site visits, fieldwork etc).

**1. Project details:**

1.1 Please provide the project title HEAG-H 12_2019: Reflexive Evidence & Systems interventions to Prevent Obesity & Non-communicable Disease (RESPOND) – economic costing and implementation evaluation

1.2 Please provide an acronym for the project (if appropriate) Action-RESPOND

1.3 Please provide the project description/protocol version number

**2. Project Team Roles & Responsibilities:**

2.1 Please provide the names, affiliations, positions and responsibilities of individuals involved in the project beyond those outlined in the HREA (e.g. technical or support staff).

N/A

**3. Resources:**

3.1 Please provide details of the resources necessary for the project to be conducted, and the funding or support being sought or secured.

Action-RESPOND is a substudy to understand the implementation phase of the RESPOND trial. The original RESPOND trial is funded through the National Health and Medical Research Council (NHMRC) Partnership Grant (APP115572, CIA Allender) which includes 12 partners including the Victorian Department of Education and Training, the Victorian Department of Health and VicHealth (see enclosed letters of support) and also funding from the Susan McKinnon Foundation.

Allender, S., Strugnell, C., Swinburn, B., Nichols, M., Peeters, A., Bell, C., Moodie, M., Orellana, L. RESPOND: Reflexive Evidence and Systems Interventions to Prevent Obesity and Non-Communicable Disease APP1151572: 2017 Partnership Projects PRC2 funding_\ commencing 2018. 2018-2023. $4,101,240 (2.6m partner contribution; $1.5m NHMRC). Primary Care Partnership and Deakin University.

Action-RESPOND is also funded through support funds provided to SY as part of a Heart Foundation Future Leader Fellowship (106654).

**4. Background:**

Please provide:

**4.1 A lay summary of the literature review (approximately 1 A4 page)**

Childhood obesity is one of the most significant global population health challenges. The World Health Organisation estimated that over 39 million children under five and 340 million children aged 5-19 years were overweight or obese in 2016. In Australia, over 25% of children aged 2-17 years are overweight or obese. It is well established that obesity is caused by a range of environmental and individual determinants that interact in complex systems.

To date, much of the empirical research aiming to prevent childhood obesity focuses on interventions that target individuals or single settings. Systematic reviews of interventions targeting single settings, such as schools and childcare centres, show that they can improve dietary outcomes, physical activity and prevent excessive weight gain, however the effects of these interventions are often small and attenuate when delivered at scale. This may be because interventions are often developed and tested in controlled research environments and therefore when scaled up, require adaptation to ensure fit within the real-world context. As such, these interventions do not maintain the same fidelity and dose when scaled up, contributing to the attenuation in effect. To address such challenges, population-level interventions that move beyond individual interventions and focus on establishing partnerships and systems, which lend themselves to ongoing and sustained lifestyle and environmental changes, are needed to meaningfully prevent obesity.

Systems thinking encompasses concepts, principles, and methodologies which allow us to understand, analyse, and address complex problems by considering them as interconnected, dynamic systems rather than isolated components. A systematic review and meta-analysis found that using system science methods in health service design and delivery significantly improved both patient outcomes (n=14 studies, OR=0.52 (95% CI 0.38 to 0.71) I2=91%) and service outcomes (n=18 studies, OR=0.40 (95% CI 0.31 to 0.52) I2=97%). Despite the promise of such interventions, there have been well-documented challenges and variability with the implementation of actions arising from these methods.

In childhood obesity prevention, the Whole of Systems Trial of Prevention Strategies for Childhood Obesity intervention (the WHO STOPS trial) applied a group model building (GMB) process to generate an agreed systems map of childhood obesity causes for a community, and in doing so, identified intervention opportunities through leveraging the dynamic aspects of the system. The WHO STOPS trial resulted in reductions in prevalence of overweight/obesity in the first two years of the intervention, however, was not sustained at four-year follow up; due in part to varying intensity of implementation.

Implementation science provides evidence-based tools, methods, and frameworks to support the design of a more systematic approach to implementation of community-developed, systems thinking-based initiatives. Intentionally embedding such methods into systems thinking approaches could provide deeper understanding into implementation context, drivers of change and help to systematically consider the evolving nature of the EBI knowledge users, systems, and organisations within community-based systems approaches . A recent systematic review however, identified just 14 studies that have described the simultaneous use of implementation science and systems science in population health prevention interventions.

**4.2 A rationale/justification (i.e. how the research will fill any gaps, contribute to the field of** research or contribute to existing or improved practice)

This pilot study seeks to address a gap identified in the literature to combine systems and implementation science approaches for obesity prevention. Given the lack of previous literature, a pilot study to provide proof of concept and feasibility data is crucial to inform the conduct of a larger trial.

**4.3 The research questions/aims/objectives/hypothesis**

The aim of this substudy is to pilot additional implementation strategies as part of the larger RESPOND study and assess the potential acceptability, feasibility and impact on implementation outcomes

**4.4 The expected outcomes**

The results will allow us to understand how to better support community-based system dynamics obesity prevention interventions.

**5. Project** **Design:**

Please provide details of:

**The research project setting**

5.1 This may include physical sites, online forums and alternatives

Health promotion teams in the 10 Local Government Areas of the Goulburn Valley and Ovens Murray Region of North East Victoria.

**6. Methodology:**

6.1 The methodological approach

 A parallel group pilot randomised controlled trial

6.2 The rationale for choices of method/s (tied to project aims/objectives)

The intent of this pilot trial is to understand the potential usefulness of implementation strategies to support participating community’s implementation identified action. This was considered the most appropriate study design to address this question.

**7. The participants including:**

7.1 A description and the number of participants

Health promotion teams (located in community health and local council) in the 10 Local Government Areas of the Goulburn Valley and Ovens Murray Region of North East Victoria

7.2 The inclusion and exclusion criteria

Those already selected to participate in the RESPOND trial. Individuals completing the survey needed to have some involvement in implementation of the identified actions from the Group Model Building process.

7.3 The sample size and statistical or power issues

As this is a pilot study, sample size was not specifically considered in this study.

7.4 Your participant recruitment strategies and timeframes (as required in addition to that outlined in the HREA)

Jan 2023- December 2024

7.5 Your approach/es to provision of information to participants and/or consent (as required in addition to that outlined in the HREA)

No amendments to the original RESPOND trial is proposed. Participants consist of those already recruited and consenting to the broader trial. For evaluation purposed, informed consent will be sought via the Qualtrics platform to participate in the evaluation.

7.6 If necessary, the type of consent provided to different participant groups, when and where, and any arrangements to confirm that consent

N/A

7.7 If necessary, details of who will be confirming or re-negotiating consent with participants and the process/es that will be undertaken

N/A

**8. Research Activities:**

What you are going to do? Please include:

8.1 The participant commitment

Participants in the intervention group will receive additional implementation strategies including a workshop, and two face to face facilitation contacts will be offered to communities randomised to receive the intervention. The strategies were developed to address key barriers to implementation and informed by the PARIHS (Promoting Action on Research Implementation in Health Services) framework. Participants will also be invited to undertake a pre and post survey at two time points (approximately July/August 2023 and May/June 2024) with all communities to evaluate the impact of implementation strategies offered to communities receiving additional implementation support. This surveys will be administered online via Qualtrics and will be deidentified. Participants will be those who have already consented to participate in the original RESPOND trial however informed consent will be sought from all participants for completion of each survey

8.2 The project duration

July 2023 to June 2024

8.3 Any participant follow-up

Post receiving implementation support (June 2024)

Please ensure your responses to Sections 9-12 comply with Deakin’s [Research Data Management procedure](https://policy.deakin.edu.au/document/view-current.php?id=23) and accurately reflect the details you have included in your [Research Data Management Plan](https://research-data.deakin.edu.au/footprints/dashboard/login?fromUrl=) (a compulsory document for all Deakin research).

**9. Data Collection/Gathering:**

9.1 What information are you going to collect/gather/generate? (as required in addition to that outlined in the HREA)

The surveys will help the research team to better understand the current impact of the RESPOND program on implementation of prevention actions identified via the Group Model Building activities. We will ask each community to nominate an appropriate individual to report on these outcomes. Measures within the survey were adapted from validated measures of fidelity, acceptability and appropriateness, reach, adoption, and cost. Additionally, all individuals in the facilitation team from those in the intervention group will also report on the acceptability and usefulness of implementation support strategy provided as part of the RESPOND project at follow up only

9.2 Data collection/gathering techniques: How will you collect/gather the information? Will any third parties be involved in any aspects of recruitment or data collection?

Data will be collected online (using the Qualtrics platform)

9.3 Impact of and response to participant withdrawal

There will be no impact of participant withdrawal. Each participant has a unique ID. Participants can withdraw at any time. Participant survey data will be removed from the aggregate analysis, if the ID is provided to the research team.

**10. Data Management:**

10.1 How will you store, provide access to, disclose, use/re-use, transfer, destroy or archive the information that you collect/gather? (as required in addition to that outlined in the HREA)

It is expected that all data collected will be via the online survey. This information is non-identifiable, and will be stored on secure, Deakin university drives for a period of five years after final publication.

**11. Data Analysis:**

11.1 How will you measure, manipulate and/or analyse the information that you collect/gather?

As this was a pilot trial, a sample size of 10 communities (with 3-7 responses in each cluster) were considered sufficient to describe the key acceptability and feasibility outcomes.

In terms of analysis, participants responses will be summed for each outcome and divided by number of items. This will provide means score each community, for each assessed construct (fidelity, acceptability, appropriateness, reach, and intention to adopt. We will undertake descriptive analysis of the constructs to describe n, %, means and standard deviations. We may also compare the responses between groups using a linear or logistic regression controlling for baseline. We will quantify overall cost required to implement the identified action.

11.2 Please describe your matching and sampling strategies

N/A- this will draw on the original response trial

11.3 Please outline how you will account for potential bias, confounding factors and missing information

N/A

11.4 Please include your statistical power calculation

As this is a pilot study, a formal sample-size calculation was not undertaken in line with best practice guidance. However, 10 communities (with between 3-8 representatives) are included, as this number was considered feasible in the timeframe and allocated resources and is sufficient to provide an adequate indication of the feasibility and acceptability of study methods and the sustainment strategy.

**12. Data Linkage:**

12.1 What linkages are planned or anticipated?

None planned

**13. Outcome measures:**

13.1 Please describe your outcome measures

The main measures collected by this trial include fidelity of implementation, intention to adopt the proposed action, acceptability and feasibility of the implementation support strategies.

**14. For research involving an unapproved therapeutic good (such as a drug, device or biological):**

14.1 Does this project involve an unapproved therapeutic good requiring a Clinical Trial Notification (CTN)? (See the [Clinical Trials webpage](https://www.deakin.edu.au/students/research/research-support-and-scholarships/integrity-secure/clinical-trials) for more information about CTNs)

Yes – go to the next question.

No – skip to Section 15 (results, outcomes and future plans)

14.2 Is Deakin intended to be the Sponsor?

Yes – go to the next question

No – skip to Section 15 (results, outcomes and future plans)

14.3 If Deakin is intended to be the Sponsor and the research requires a Clinical Trial Notification (CTN), has the CTN, Clinical Trial Sponsorship Request Form and Protocol been submitted to [research-integrity@deakin.edu.au](mailto:research-integrity@deakin.edu.au) for assessment?

Yes – assessment completed and the CTN must now be submitted to the Therapeutic Goods Administration (TGA) by Deakin (as Sponsor). Please attach evidence of assessment and the CTN form. You will be contacted by the Human Research Ethics Office regarding submission of the CTN to the TGA.

If not, please submit the draft CTN, Clinical Trial Sponsorship Request Form and Protocol to [research-integrity@deakin.edu.au](mailto:research-integrity@deakin.edu.au) for assessment before submitting this application to DUHREC. See the [Clinical Trials webpage](https://www.deakin.edu.au/students/research/research-support-and-scholarships/integrity-secure/clinical-trials) for further information. The Clinical Trial Sponsorship Request Form can be requested by contacting [research-integrity@deaknin.edu.au](mailto:research-integrity@deaknin.edu.au).

14.4 What is/are the drug(s) and/or device(s):

- Approved name
- Trade name (if any)
- Manufacturer
- Supplier of drug/device (e.g. manufacturer/pharmacy)
- Approved therapeutic indication, dosage/duration in Australia
- Believed mode of action
- Dosage regimen
- Mode of excretion
- Known adverse events
- Known contra-indications or warnings
- If arrangements have been made for a Pharmacy Department to receive or dispense the drugs involved in this project, explain how the drugs will be received and dispensed for the purposes of the research project

**15. Results, Outcomes and Future Plans:**

15.1 Please outline your plans for return of results of research to participants – include an ethically defensible plan in accordance with National Statement [3.1.65](https://nhmrc.gov.au/about-us/publications/national-statement-ethical-conduct-human-research-2007-updated-2018#toc__438) or [3.2.15](https://nhmrc.gov.au/about-us/publications/national-statement-ethical-conduct-human-research-2007-updated-2018#toc__725) or [3.3.36-3.3.61](https://nhmrc.gov.au/about-us/publications/national-statement-ethical-conduct-human-research-2007-updated-2018#toc__826), as appropriate.

We will provide a summary to study participants upon request. We will also present all findings at key network meetings following the research

15.2 Please describe your plans for dissemination and publication of project outcomes

- Oral Presentations, and PowerPoint slides to RESPOND Partners (circulated post network meetings)

- Peer review journal publications

- Scientific conferences

15.3 Please list other potential uses of the data at the end of the project

N/A

15.4 Please detail the project closure processes

At the conclusion of the project and after all the final reports/publications etc have been published, the electronic data will remain on the restricted access GLOBE database on the Deakin servers for 5-years post-publication. We will consult Deakin Archives after the 5-year period has expired e.g. 2028 on how to appropriately destroy this electronic data. If the lead investigator (Prof Allender) should leave Deakin University we will make appropriate arrangements to transition this responsibility to another named researcher on the HREA application or Head of School (where appropriate). 15.5

Please outline your plans for sharing and/or future use of data and/or follow-up research

We don’t plan to share the data to any other research group or agency. There is no planned follow-up research at this stage

15.6 Please describe any anticipated secondary use of data

NA
