# Supplementary material for: A protocol of a pilot randomised trial (Action-RESPOND) to support rural and regional communities with implementing community-based systems thinking obesity prevention initiatives
Source: PLoS One. 2024 May 2;19(5):e0302047. doi: 10.1371/journal.pone.0302047 (PMC11065269; doi:10.1371/journal.pone.0302047)
Supplement: Attachment — Submitted filename: A-RESPOND protocol_P One R1_response to reviewers.docx [file pone.0302047.s003.docx]

20/02/2024.

Dear Editor,

We thank the reviewers for their review of our manuscript submitted to PLOS one**: A protocol of a pilot randomised trial (Action-RESPOND) to support rural and regional communities with implementing community-based systems thinking obesity prevention initiatives.**

Please find attached our point-by-point response (in blue italics) to reviewer comments and a revised manuscript with all changes highlighted in the manuscript using track changes.

**JOURNAL COMMENTS**

**1. Please ensure that your manuscript meets PLOS ONE's style requirements, including those for file naming. The PLOS ONE style templates can be found at** 
[**https://journals.plos.org/plosone/s/file?id=wjVg/PLOSOne_formatting_sample_main_body.pdf**](https://journals.plos.org/plosone/s/file?id=wjVg/PLOSOne_formatting_sample_main_body.pdf) **and** 
[**https://journals.plos.org/plosone/s/file?id=ba62/PLOSOne_formatting_sample_title_authors_affiliations.pdf**](https://journals.plos.org/plosone/s/file?id=ba62/PLOSOne_formatting_sample_title_authors_affiliations.pdf)

*We have double checked out manuscript to ensure alignment with journal requirements.*

**2. We note that the grant information you provided in the ‘Funding Information’ and ‘Financial Disclosure’ sections do not match.**

**When you resubmit, please ensure that you provide the correct grant numbers for the awards you received for your study in the ‘Funding Information’ section.**

*We have checked and confirm that this is now aligned.*

**3. Thank you for stating the following financial disclosure: 
"RESPOND is funded through the National Health and Medical Research Council (NHMRC) (APP115572, CIA SA), VicHealth, Nexus Primary Health, Goulburn Valley Primary Care Partnership and Deakin University. Action-RESPOND is also funded through support funds provided to SY as part of a Heart Foundation Future Leader Fellowship (106654). The opinions, analysis, and conclusions in this paper are those of the authors and should not be attributed to the NHMRC, the Victorian Department of Health or the Victorian Department of Education and Training. SY is supported by a Heart Foundation Future Leader Fellowship (106654). The participating communities were not provided with additional funding to implement the actions."**

**Please state what role the funders took in the study.  If the funders had no role, please state: "The funders had no role in study design, data collection and analysis, decision to publish, or preparation of the manuscript." 
If this statement is not correct you must amend it as needed. 
Please include this amended Role of Funder statement in your cover letter; we will change the online submission form on your behalf.**

*We have added the relevant statement indicating that funders have no role in the study.*

**4. Thank you for stating the following in the Competing Interests section: 
"S.A. is a co-inventor of the STICKE software, which is used in this study. All other authors have no conflict of interest to declare. "**

**Please confirm that this does not alter your adherence to all PLOS ONE policies on sharing data and materials, by including the following statement: "This does not alter our adherence to PLOS ONE policies on sharing data and materials.” (as detailed online in our guide for authors** [**http://journals.plos.org/plosone/s/competing-interests**](http://journals.plos.org/plosone/s/competing-interests)**).  If there are restrictions on sharing of data and/or materials, please state these. Please note that we cannot proceed with consideration of your article until this information has been declared.**

**Please include your updated Competing Interests statement in your cover letter; we will change the online submission form on your behalf.**

*We can confirm this does not alter adherence to the PLOS one policies and have added the suggested statement as requested.*

**5.PLOS requires an ORCID iD for the corresponding author in Editorial Manager on papers submitted after December 6th, 2016. Please ensure that you have an ORCID iD and that it is validated in Editorial Manager. To do this, go to ‘Update my Information’ (in the upper left-hand corner of the main menu), and click on the Fetch/Validate link next to the ORCID field. This will take you to the ORCID site and allow you to create a new iD or authenticate a pre-existing iD in Editorial Manager. Please see the following video for instructions on linking an ORCID iD to your Editorial Manager account:** [**https://www.youtube.com/watch?v=_xcclfuvtxQ**](https://www.youtube.com/watch?v=_xcclfuvtxQ)

*The corresponding author has now linked their ORCID ID in the Editorial Manager.*

**6. We note that the original protocol that you have uploaded as a Supporting Information file contains an institutional logo. As this logo is likely copyrighted, we ask that you please remove it from this file and upload an updated version upon resubmission.**

*This is removed for the document RESPOND monitoring project-protocol DU-HREC. All other institutional logos are part of funding submission and ethics approval processes and thus cannot be edited.*

**REVIEWER COMMENTS**

**Reviewer #1:**

**Thank you for the opportunity to review this paper.
My review mainly concerns only the statistical aspects of the study.
The paper is well write and the simple statistical analysis plan well described**

*Thank you for your review of our manuscript. We have updated the statistical analysis section in response to reviewer’s 2 comments.*

**Reviewer #2:**

**Thank you for the opportunity to review this paper describing the protocol of a randomized controlled trial to address child obesity in Victoria Australia. I commend the authors for developing a well-written manuscript and for their important efforts to address childhood obesity. While the manuscript is well-written and addresses a very important topic, there are several areas where the manuscript can be strengthened, which may also have implications for the Action-RESPOND trial. I offer the following suggestions below to strengthen the manuscript and its contribution to the literature. I hope the authors find my feedback to be helpful and constructive**.

*Thank you for your helpful and constructive feedback. We have attempted to address the comments below and agree that it has helped improve the manuscript.*

**Title
1. As written, the title is rather lengthy. Can the title be shortened a bit?**

*Thank you for the suggestion – we have shortened the title.*

*A protocol of a pilot randomised ~~controlled~~ trial (Action-RESPOND) to support rural and regional communities ~~in Victoria, Australia~~, with implementing community-based systems thinking ~~child~~ obesity prevention initiatives.*

**Abstract
2. In the methods section of the abstract, should the first mention of “(Action-RESPOND”) appear after the words “multicomponent implementation strategy…”? It’s a bit confusing to figure out of the intervention is the implementation strategy or community-based systems thinking. Based on how the rest of this section is written, it seems like Action-RESPOND is the implementation strategy. Therefore, consider moving the first mention of “(Action-RESPOND)” next to the words “multicomponent implementation strategy.”**

*We agree that Action-RESPOND is the implementation strategy and have moved it to the appropriate section of the abstract.*

**3. I know words will be tight, but would it be possible to briefly list the 7 implementation strategies of Action-RESPOND in the methods section?**

*Thank you this suggestion, we have added this to the methods section of the abstract:*

*Implementation strategies aimed to ensure initiatives implemented are i) evidence-based, ii) address community’s specific needs and iii) are suitable for local context. Strategies also aimed to increase the community’s capacity to implement, through iv) improving the health promotion team’s implementation knowledge and skills, fostering v) leadership, vi) physical resources and vii) community culture to drive implementation.*

**Introduction**
**4. Line 58: As part of the opening sentence, consider mentioning some of the health conditions associated with childhood obesity to highlight the point that obesity is a population health challenge.**

*We have added the following to the introduction:*

*Childhood obesity is one of the most significant global population health challenges, as it increases children’s risk of developing type 2 diabetes, cardiovascular diseases, and liver complications, during their childhood as well as their adulthood [1].*

**5. Lines 63-64: Provide some context about how environmental and individual determinants interact in complex systems to cause obesity. Stating some specific examples would help show readers how the intervention is addressing some of the specific environmental and individual determinants.**

*We have added the following to the introduction:*

*It is well established that obesity is caused by a range of environmental and individual determinants that interact with complex systems [5, 6]. For example, broader environmental determinants such as a lack of play spaces or walking paths is likely to reduce an individual’s motivation to engage in physical movement and activity, while availability and marketing of ‘less healthy’ foods could result in poor dietary choices [1].*

**6. Lines 76-80: Are there any specific examples that can be provided about effective partnerships and systems lending to ongoing and sustained lifestyle and environmental changes? While the contents in this paragraph sound good, it would be good to provide a tangible example of something that worked well to address obesity.**

*Thank you for suggesting this, we have added the following example to the introduction:*

*… which lend themselves to ongoing and sustained lifestyle and environmental changes, are needed to meaningfully prevent obesity [13]. The "Shape Up Somerville" program in Massachusetts (USA) is one such example of where community-based systems level changes has produced meaningful changes over time [16]. This program involved collaboration between government agencies, schools, local businesses, and community organizations to implement multi-level interventions targeting nutrition, physical activity, and built environment changes between 2003 and 2005. This program produced positive changes on child and parent BMI post intervention with an estimated $197,120 USD net benefits over 10-years. The authors postulated that this positive outcome, which extended beyond improvement to individual children’s health, was made possible by the community-wide, systems-based nature of the interventions.*

**7. Lines 108-113: The authors talk about implementation science providing evidence-based tools, methods, and frameworks that “intentionally embedding such methods into systems thinking approaches…” It would be helpful to provide some specific examples of these tools and methods so that readers can have a tangible idea about what could specifically be embedded into systems thinking approaches. Also, providing some specific examples in the introduction section would help set a better context for the aims that are listed in lines 122-124.**

*We have added the following elaboration to the manuscript:*

*Implementation science [30] provides evidence-based tools, methods, and frameworks to support the design of a more systematic approach to implementation [30] of community-developed, systems thinking-based initiatives.* *In particular, implementation science draws on empirically-developed frameworks to guide the systematic planning, implementation and evaluation of complex interventions. For example, frameworks such as the Consolidated Framework of Implementation Research [31] and the Theoretical Domains Frameworks [32] have been widely used to systematically identify barriers to implementation and support the selection of evidence-based implementation strategies to improve implementation processes [33].*

**MATERIALS AND METHODS**
**8. Line 135: The authors should specify the criteria that were used to determine the rural nature of the communities (i.e., how do the investigators know that an area is rural?).**

*This classification was based on that determined by the Victorian government using the Australian Statistical Geography Standard Remoteness Structure, which considers factors including population size, geographic location and access to services.*

*The following explanation has been added to the manuscript:*

*Rural and regional classifications were based of that specified by the Victorian Department of Health [37] which is based on the Australian Statistical Geography Standard Remoteness Structure.*

**9. Line 143: Please give some examples of “key partners.”**

*We have added the list of partners to the manuscript (line 169 – 173).*

**10. Line 147: Are there any results that can be reported regarding the RESPOND program? Perhaps an outcomes paper that can be cited? It would be helpful to know about the effectiveness of RESPOND to support why there is a need to build upon this program (e.g., was the program ineffective, therefore warranting Action-RESPOND?).**

*RESPOND was undertaken between 2020-2023 and coincided with COVID 19 and bush fires in the region. Although the program continued to be delivered to communities, implementation and primary outcome data collection was disrupted due to some of the disruptions faced during this time. Action-RESPOND provided an opportunity for communities to more explicitly focus on implementation following such challenges. We have added the following to the manuscript.*

*All ten communities received the RESPOND program between 2020 – 2023. The delivery of the RESPOND program coincided with significant disruptions due to COVID-19 and bush fires in the region, significantly impacting on organisational priorities, loss of momentum of implementation, and redeployment of human resources [24].*

**11. Lines 148-157: Can the authors speak to how they are going to assess if communities have naturally adopted some of the enhanced implementation support that is being provided in Action-RESPOND?**

*We will assess using a survey at follow up whether any contamination occurred (i.e. whether any communities in the control group also received additional implementation support). See “Potential co-intervention and contamination” under “outcomes”.*

**12: Lines 164-167: As I mentioned previously, please provide the criteria that were used to determine whether communities were indeed rural.**

*Please see response to comment #8.*

**13. Line 234: Will the investigators be keeping track of the requests for local tailored support? This information will be helpful for understanding how much support is needed to incorporate implementation strategies.**

*Thank you for flagging this. Yes, the implementation coach will keep track of all contact points with the Implementation Champion, including the duration and nature of support provided, using an excel spreadsheet. The following has been added in Line 401 - 403:*

*The Implementation Coach is available to provide additional local tailored support throughout the intervention period. All additional support provided including each contact, attendees, duration and nature of support will be captured using a Microsoft Excel spreadsheet.*

**14. Lines 239-241: I am a little confused as to how the implementation coach is situated within the targeted communities. Is this saying that the implementation coach lives in these communities? Since there are 10 distinct rural communities, how is it possible for one implementation coach to be situated in all of them? Consider clarifying the implementation coach’s relationship with these communities.**

*Thank you for bringing this up. You are right in that the implementation Coach is located in one of the ten communities that is located more broadly in the Ovens Murray region of Victoria. We have clarified this:*

*The Implementation Coach (MH) has 10 years of health promotion experience working within the targeted communities and is situated within one community of the Ovens-Murray region.*

**15. Line 379: the authors mention that follow-up will occur at approximately 9-months post-intervention. What are the implications of having a 9-month follow-up but having participants set goals that are practical and feasible to achieve in the next 9-12 months (line 321)?**

*Thank you for bringing this up. This was a typo in the manuscript, the communities were asked to develop goals for the next 6-9 months. We have changed this in Line 359.*

**16. Line 381: In this section, it would be helpful to see the actual items that are being used to assess feasibility and adoption. Further, I would recommend adding a bit more information about the scale other than the response options ranging from 1-7. For example, what does a response option of 1 indicate compared to a response of 7?**

*Thank you for this, we have added information on the response options for the survey items, for all outcomes that are assessed by surveys (Line 422 – 458).*

**17. Lines 393-394: Please give just a little more context about the response options of the Likert scale (similar to what the authors did on line 400).**

*Thank you for this, we have added information on the response options for the survey items, for all outcomes that are assessed by surveys (Line 422 – 458).*

**18. Lines 433-434: Please add a citation to support that it is a best practice to not conduct a formal sample-size calculation for pilot studies (Note: I agree with this, but it would be helpful for others who question this way of thinking).**

*Thank you for highlight this, we have added the following citation: Eldridge et al. 2016. CONSORT 2010 statement: extension to randomised pilot and feasibility trials.*

**19. Line 438: In the data management section: Do the authors have a plan to assess for data entry errors and the process they plan to take to rectify potential errors?**

*Additional detail regarding handling of possible errors have been added to the data management section (Line 493 – 503).*

**20. Line 448 (Data analysis section): I’m struggling to understand how a linear regression can be used to compare fidelity and adoption between the two groups. The control group will not be incorporating the new implementation strategies, correct? Can the authors explain this?**

*The measures of fidelity and adoption relate to implementation of one selected obesity prevention ‘action’ selected by the community, not of the implementation strategy. As such the linear regression will allow for comparisons to be made between the intervention and control groups, regarding the extent in which the selected obesity prevention action was adopted and implemented as intended. This will provide some insights into the potential usefulness of the implementation strategies included within Action-RESPOND on action implementation. This has been clarified in text:*

*For implementation outcomes (fidelity and adoption of the selected action for implementation), item responses will be summed to provide an overall fidelity and an overall adoption score for both the intervention and control group.*
 **21. Line 463: The authors mention that the advisory group will assess suitability for progression to a fully powered trial in similar communities based on data on fidelity, adoption, and acceptability of the implementation strategies. I am curious as to which outcome the authors will use for advancing to a fully powered trial. For a fully powered trial, it seems like the authors would want to power around weight reduction/obesity prevention rather than indicators of fidelity, adoption, and acceptability of implementation strategies. In other words, it will be important to know if the authors can actually make improvements in obesity before moving forward with a fully powered trial.**

*Thank you for your comments, the advisory group will also draw from systematic reviews of the impact of community-based obesity prevention studies to provide an understanding of the impact of such approaches on child level outcomes. Given this study focuses on understanding the impact of implementation strategies on action implementation, the data collected by this study together with the broader systematic review data will allow for assessment on the potential for a larger trial focused on implementation specifically. We have elaborated on this in the manuscript:*

*The advisory group for this research will assess suitability for progression to a fully powered trial in similar communities in Victoria, using data collected related to fidelity, adoption, and acceptability of the implementation strategies via this study as well as systematic reviews of systems-based obesity prevention interventions on the potential benefits on child outcomes. Together this data will allow for assessment on the potential for a larger trial focused on implementation specifically.*
 **22. Line 491: A citation is needed for the statement “Despite calls to better understand…”**

*We have added two citations for this statement:*

I. *Whelan et al. 2023. Combining systems thinking approaches and implementation science constructs within community-based prevention: a systematic review.*
II. *Swinburn et al. 2019. The global syndemic of obesity, undernutrition, and climate change: the Lancet Commission Report*

**23. As I read the discussion section, I found myself wondering how RESPOND actually addressed childhood obesity. I went back to the methods section to read about RESPOND (lines 133-147). It looks like there was routine childhood obesity and risk factor monitoring and the implementation of strategies identified in the GMB processes. It would be helpful to talk more about RESPOND in the methods section. In particular, what strategies were actually implemented to address childhood obesity?**

*Although RESPOND supports communities with identifying leverage points within the system for action as part of the GMB process, it provides limited targeted implementation support to communities. A range of actions are identified as part of the GMB processes however the extent of implementation of each of the actions is not explicitly supported as part of the RESPOND study. This has resulted in some variability in actual implementation process between the communities. The intent of Action-RESPOND is to use best practice implementation science methods to design and provide more consistent implementation support to communities. We have included some examples of action ideas that arose from GMBS in the Methods section (Line 241 to 244).*

We hope that we have adequately addressed the reviewer comment and look forward to hearing regarding next steps.

Regards,

A/Prof Serene Yoong.
